# Socio-demographic and lifestyle factors associated with multimorbidity in New Zealand

**DOI:** 10.4178/epih.e2020001

**Published:** 2019-12-27

**Authors:** Nayyereh Aminisani, Christine Stephens, Joanne Allen, Fiona Alpass, Seyed Morteza Shamshirgaran

**Affiliations:** 1 Department of Epidemiology and Statistics, Faculty of Health Sciences, Neyshabur University of Medical Sciences, Neyshabur, Iran; 2 Healthy Aging Research Centre, Neyshabur University of Medical Sciences, Neyshabur, Iran; 3 School of Psychology, Massey University, Palmerston North, New Zealand

**Keywords:** Multimorbidity, Demographic factors, Lifestyle, Incidence, New Zealand

## Abstract

**OBJECTIVES:**

The incidence of multimorbidity (MM) and its correlates among older adults remain poorly understood. This study aimed to examine the socio-demographic and lifestyle factors associated with MM in New Zealand.

**METHODS:**

People aged 55-70 years were invited to participate in a population-based cohort study, the Health Work and Retirement Study, in 2006. Those who accepted the invitation and completed the baseline questionnaire were followed up on a biennial basis. Data on socio-demographic factors, health and lifestyle behaviours, and diagnoses of chronic diseases were obtained from baseline and 6 waves of follow-up. Generalised estimating equations (GEE) adjusted for both time-constant and time-varying factors were used to model factors associated with the onset of MM.

**RESULTS:**

A total of 1,673 participants (with 0 or 1 chronic condition) contributed to an overall 8,616 person-years of observation. There were 590 new cases of MM over 10 years of follow-up, corresponding to an overall incidence of 68.5 per 1,000 person-years. The results of the age- and sex-adjusted GEE analysis showed that age, ethnicity, living alone, obesity, hypertension, and having 1 chronic condition at baseline were significant predictors of MM onset. Higher education, income, physical activity, and regular alcohol consumption were protective factors. In a fully adjusted model, marital status (odds ratio [OR], 1.18; 95% confidence interval [CI], 1.01 to 1.37; p=0.039), hypertension (OR, 1.23; 95% CI, 1.02 to 1.48; p=0.032) and having 1 chronic condition at baseline (OR, 2.92; 95% CI, 2.33 to 3.67; p<0.001) remained significant.

**CONCLUSIONS:**

The higher incidence of MM among Māori people, socioeconomically disadvantaged groups, those with low physical activity, and obese individuals highlights the importance of targeted prevention strategies.

## INTRODUCTION

Multimorbidity (MM), defined as the “coexistence of 2 or more chronic diseases in the same individual,” is a current health concern due to population ageing [1]. The complex health needs created by MM pose global health challenges for individuals and health care systems, with adverse effects on health outcomes, costs, and resources [2,3]. MM has been reported to be associated with a poor quality of life [4], increased health care costs and utilisation [5], functional decline [6], and death [7] by a wealth of cross-sectional studies that have investigated the prevalence and predictors of MM among older adults. However, the incidence of MM and its correlates among older adults remain poorly understood [8].

Publications on this topic using a prospective or longitudinal approach are scarce. A Swedish study [9] including 418 older persons (78+ years) investigated predictors of incidence of MM, and a Chinese study [10] was conducted among approximately 1,000 individuals 20 years of age and older to assess the associations between nutritional factors and MM. A Finnish study [11] included approximately 33,000 males and females 25-64 years of age, aiming to investigate associations between lifestyle and clinical factors and the onset of MM. We found 2 reports based on data from different waves of the English Longitudinal Study on Ageing (ELSA) among persons aged 50 and up [12,13]; one aimed to examine the relationship between physical activity (PA) and the incidence of MM (n=15,688), and the other aimed to assess lifestyle factors in relation to the development of MM (n=10,518).

Although these studies provide important information about the predictors of MM, limitations in the literature to date include a focus on people over 78 years of age [9], an operational definition of MM based on a limited number of chronic diseases [4,11], and a focus on more specific topics [10,12,13].

We aimed to investigate the incidence of MM (in a population without MM at baseline) and to examine its associations with important socio-demographic, lifestyle, and clinical factors based on data from 6 waves of a nationwide longitudinal study on ageing in New Zealand.

## MATERIALS AND METHODS

### Study population

The Health, Work and Retirement Study is a biennial prospective cohort study of community-dwelling older adults. The questionnaire form assesses domains of health and wellbeing, family and social support, work and retirement, financial wellbeing, and cultural identity. The study commenced in 2006 as a postal survey of a representative national sample of community-dwelling adults aged 55-70, who were randomly selected from the New Zealand electoral roll. The original survey had a response rate of 53% (n= 6,662), of whom 2,632 consented to be invited to participate in subsequent waves. Of this subsample, 1,609 (41%) were lost to follow-up over the subsequent 5 waves (n=212 to death, and the remaining unknown). For the current analysis, the baseline study population (at-risk population) were participants who both consented to follow-up and did not meet the criteria for MM at baseline. The age and sex distribution of the original responders, baseline cohort, and the study participants were similar.

### Measures

Information on a range of demographic, socioeconomic, lifestyle, and clinical factors obtained from core survey measures were examined in the current analysis.

#### Multimorbidity

The outcome variable was the onset of MM based on participants’ responses to a question asking, ‘Has a doctor, nurse or other healthcare worker told you that you have any of the following health problems?’, which preceded a list of chronic conditions. Nine groups of diseases assessed in all survey waves were included in the current analysis: heart diseases, stroke, other neurologic diseases (epilepsy, Parkinson disease, migraine headache, Alzheimer disease/dementia), musculoskeletal (arthritis, osteoporosis, 2hip/knee replacement), diabetes mellitus, respiratory diseases (chronic obstructive pulmonary disease, asthma), chronic liver conditions (cirrhosis), cancer, and mental disorders (depression, anxiety, and other mental diseases). In all waves, MM was defined as reporting a positive diagnosis for 2 or more groups of chronic diseases.

#### Demographic variables

Age, which was provided as a continuous variable, was also categorised as <65 years and ≥65 years. Marital status was dichotomised as married/living with a partner or divorced/separated/single/widow; ethnicity was classified as the priority ethnic groups in New Zealand, Māori or non-Māori (Europeans, Asians, Pacific people, and other ethnicities). Socioeconomic status indicators included the highest educational qualification (categorised as no secondary, secondary, post–secondary, or tertiary education) and annual personal income (≤25,000, 25,001-50,000, 50,001-70,000, >70,000 New Zealand dollar [NZ$]). For multivariate statistical modelling, annual income was categorised into 3 levels (≤25,000, 25,001-50,000, and >50,000 NZ$).

#### Health behaviours

Smoking was defined by a question that asked respondents to identify themselves as regular smokers or not. Alcohol consumption was measured by a question that assessed the frequency of drinking, which was classified into 2 categories: regular alcohol consumption (≥2 drink/wk) and non-regular alcohol drinking (≤1 drink/wk). PA was measured by the frequency of moderate/brisk walking or vigorous activity in the last 7 days and categorised into 2 levels: ≥2 times/wk (sufficient) or 1 times/wk/none (insufficient).

#### Clinical variables

Hypertension was considered as a dichotomous variable (yes/no) based on participants’ responses to a question about diagnosed health problems. Body mass index (BMI; weight in kilograms divided by height in meters squared) was measured in the 2008 survey wave and was categorised as normal weight (<25.0 kg/m^2^), overweight (25.0-29.9 kg/m^2^), and obese (≥30.0 kg/m^2^).

### Statistical analysis

The statistical significance of differences in the characteristics of people with and without 1 chronic condition at baseline was determined by using the Student t-test for continuous variables and the chi-square test for categorical variables. Incidence rates (IRs) and 95% confidence intervals (CIs) of MM per 1,000 person-years were calculated with stratification by age, sex, and ethnicity. Generalized estimating equations (GEEs) with an exchangeable correlation matrix and robust standard errors were used to assess the factors associated with the onset of MM. We calculated the odds ratios (ORs) and 95% CIs for MM by socio-demographic and lifestyle factors, and constructed 3 models: crude, adjusted for age and sex, and fully adjusted. The fixed covariables were sex, ethnicity, education, income, and BMI. All other variables were considered as time-varying.

All estimates were reported with 95% CIs and a significance level of 0.05. All analyses were performed using the Stata version 14 (StataCorp., College Station, TX, USA), except the IR calculations, which were run using OpenEpi [14].

### Ethics statement

The study procedures were approved by the Massey University Human Ethics Committee (MUHEC Southern B 09/70).

## RESULTS

Of the 2,632 participants who responded to the baseline questionnaire and agreed to participate in follow-up, 957 met the criteria for MM at baseline and were excluded from the analysis. There were 1,673 respondents who reported either no disease (n=776) or 1 disease (n=897), of whom a total of 590 developed MM over the follow-up period (Supplementary Material 1).

Table 1 shows the baseline characteristics of the study participants who reported 0 or 1 chronic condition. Relative to those with no chronic conditions, participants with 1 condition at baseline were older (p=0.009) and less educated (p=0.018), and had a lower annual income (p<0.001), a higher frequency of overweight/obesity (p=0.040), and a higher frequency of hypertension (p< 0.001). The frequency of regular alcohol consumption was lower among those with 1 chronic condition than among their counterparts with no chronic conditions (p=0.001).

**Figure 1.**
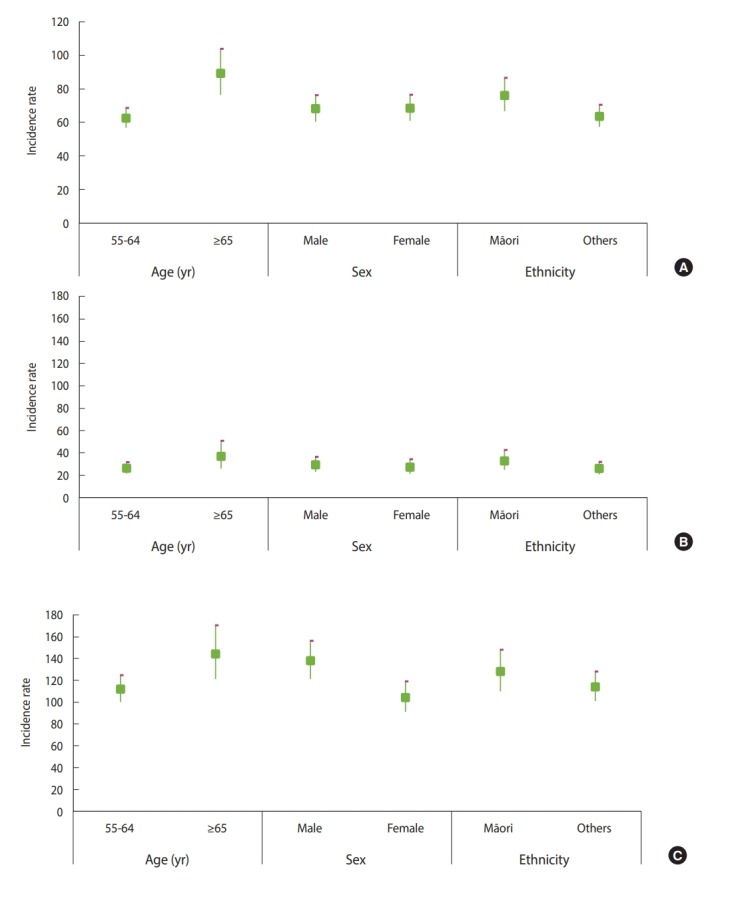
Incidence rates of multimorbidity by age, sex, and ethnicity strata among adults (A) aged 55 and over, 2006-2016, (B) without a chronic condition at the baseline, 2006-2016, and (C) with 1 chronic condition at the baseline, 2006-2016, in New Zealand.

A total of 1,673 participants (with 0 or 1 chronic condition) contributed to an overall 8,616 person-years of observation. There were 590 new cases of MM over 10 years of follow-up, which resulted in an overall incidence of 68.5 per 1,000 person-years. The incidence was higher among older participants than among their younger counterparts (89.6 vs. 62.7 per 1,000 person-years, respectively; p<0.001), but did not differ significantly by sex. However, Māori participants had a higher incidence of MM than non-Māori participants (76.4 vs. 63.9 per 1,000 person-years respectively, p=0.04) (Figure 1A). The incidence of MM was further stratified for demographic groups by the presence (Figure 1C) or absence of 1 chronic condition at baseline (Figure 1B). In all groups, the incidence of MM was significantly higher among those with 1 chronic condition than among those with no chronic conditions.

The results of the GEE analysis adjusting for age and sex (Table 2) indicated that older age, Māori ethnicity, living alone, obesity, hypertension, and having 1 chronic condition at baseline were significant predictors of MM onset. PA, regular alcohol consumption, completion of post-secondary or tertiary education, and an annual income greater than 50,000 NZ$ were protective predictors of MM onset. The incidence of MM was inversely associated with higher income and education. In a fully adjusted model (data not shown), only marital status (OR, 1.18; 95% CI, 1.01 to 1.37; p=0.039), hypertension (OR, 1.23; 95% CI, 1.02 to 1.48; p=0.032), and having 1 chronic condition at baseline (OR, 2.92; 95% CI, 2.33 to 3.67; p<0.001) remained significant predictors of MM onset.

## DISCUSSION

This study examined the incidence of MM and its socioeconomic and lifestyle predictors using data from a prospective cohort study of older New Zealand adults. The overall incidence of MM over 10 years of follow-up was 68.5 per 1,000 person-years. This rate is higher than the reported IR of 59 per 1,000 person-years based on data from different waves of the ELSA [12]. However, it is lower than the IR reported by a Swedish study [9], which found an IR of 126 per 1,000 person-years in those without any diseases at baseline and 329 per 1,000 person-years among those with 1 chronic condition at baseline. However, there are several reasons why the comparison of IRs across studies is problematic, as variation in reported IRs may reflect differences in the classification of MM, length of follow-up, participants’ age, or health related attrition.

For example, MM may have been less frequently observed in the current study than in the Swedish study by Melis et al. [9], because the participant cohort was younger (55-70 years at recruitment vs. people aged 78+ years old). Further, Melis et al. [9] used multiple sources to verify diagnoses, whereas in the current study, MM was based on self-reported diagnoses of chronic conditions by doctors or health professionals. The age distribution of study participants and the classification of MM based on self-reported conditions are similar between our study and the ELSA study [12]; however, the list of chronic conditions used differed. Other factors could also contribute to these differences, such as ethnic variation and diversity in lifestyle and socioeconomic factors across populations; however, these factors are difficult to quantify in light of these common methodological variations.

The major strength of the present study is that it examined socio-demographic, health behaviour, and lifestyle factors associated with the onset of MM in an older New Zealand population sample. The analyses of the present study indicate that the incidence of MM was higher among older participants than among their younger counterparts (89.6 vs. 62.7 per 1,000 person-years, respectively), which is in line with evidence that the incidence of MM is higher among older adults [9,12]. In the age- and sex-adjusted models, ethnicity, marital status, education, and income were associated with MM. Māori participants had a higher incidence of MM than non-Māori participants (76.4 vs. 63.9 per 1,000 person-years), which is in line with the findings of a Canadian study that observed a higher prevalence of MM among indigenous populations than among non-indigenous populations [15]. These findings indicate that health care and promotion should carefully consider appropriate targeting of services and messages to improve the health of older members of indigenous populations. As in the current investigation, socioeconomic factors protective against MM have also been assessed in other studies [15-17], which have found financial hardship during childhood [16], low education [17], and living in the lowest income quintile [15] to be associated with MM. A higher education level was similarly associated with a lower IR of MM in a Swedish study [9].

Among the lifestyle factors that were investigated, obesity and insufficient PA were significant predictors of MM onset in age and sex-adjusted models. We found that regular alcohol consumption was a protective predictor of MM onset. No significant association between smoking and MM was found in our analysis. The current finding regarding the protective effects of PA was similar to the findings of other studies [11-13,15] , which showed an inverse association between PA and MM. However, the effect of PA was attenuated in the fully adjusted model, which might be explained by the fact that those with 1 chronic condition at baseline tended to have inadequate PA. Evidence has shown that adherence to PA is low in individuals with chronic diseases [18]. Our findings on smoking and alcohol consumption differed from those of a Finnish study [11] which reported that smoking and alcohol consumption increased the risk of MM. The data based on the ELSA study [12] are in line with the findings of our study for smoking, but contrast with those of our study in regard to excess alcohol consumption. A Canadian study also did not find any association between excess alcohol consumption and MM [15]. Risky alcohol consumption is associated with a higher risk of many chronic conditions, and in people with existing chronic diseases it is related to a higher risk of medical conditions [19,20]. An explanation for our results might be that those with 1 chronic condition at baseline did not tend to use alcohol regularly. In our study, obesity was negatively related to MM occurrence; however, in other studies, a more pronounced association has been found in combination with physical inactivity or inadequate fruit and vegetable intake [12]. We also found that hypertension and having 1 chronic condition at baseline were strongly associated with the onset of MM and that these relationships remained significant in the fully-adjusted model. Previous cross-sectional studies have also reported a strong association between high blood pressure and MM [15,21]. The role of hypertension has been conceptualised differently, with some studies considering hypertension as a chronic condition [9,12] and others not including hypertension [21]. Due to the high prevalence of high blood pressure in older adults, the prevalence or incidence of MM might be markedly increased if this condition is considered in the definition of MM. In our analysis, those who had 1 chronic condition were more likely to develop MM, which is in line with the findings of a Swedish study [9] that the IR of MM was higher among those with 1 chronic condition at baseline.

The value of this study lies in its longitudinal nature and the provision of additional insights into the burden of MM and its correlates. However, we acknowledge that our results might be particularly vulnerable to biases associated with the assessment and operational definition of MM. This is a particular threat to comparison between studies. The current study employed a list of doctor/health professional–diagnosed self-reported chronic conditions to identify MM. While this is a common method to ascertain MM in the literature, heterogeneity in this definition and in the number of chronic conditions included remains an issue. In addition, in this analysis, only current smoking and alcohol consumption were considered, since we did not have further details on these variables.

This project is the first longitudinal study providing evidence on the incidence of MM and its associations with socioeconomic and lifestyle factors in New Zealand, and the results may be of particular interest for public health policy. The higher incidence of MM among Māori, socioeconomically disadvantaged groups, those with inadequate PA, and obese individuals highlights the importance of targeted information and associated prevention strategies. The strong association between hypertension and having 1 chronic condition at baseline with the incidence of MM should be considered as part of care and management strategies.

Further research is recommended using a better definition of MM and including more details on smoking and alcohol consumption to be able to show to what extent quitting smoking or alcohol consumption might be related to the incidence of MM. In addition, the role of hypertension in the development of MM might of interest for further research, as we found a significant association between the incidence of MM and hypertension. Therefore, it would also be useful to explore the pattern of chronic conditions related to hypertension.

## Figures and Tables

**Table 1.** Baseline characteristics of adults 55 years of age and older according to the presence of a chronic condition

Characteristics (2006)	Chronic condition		p-value
	Without	With 1	
Age (yr)			0.009
55-64	618 (79.6)	666 (74.3)	
≥65	158 (20.4)	231 (25.8)	
Sex			0.255
Female	391 (50.4)	477 (53.2)	
Male	385 (49.6)	420 (46.8)	
Education			0.018
No secondary	180 (23.4)	261 (29.4)	
Secondary	234 (30.4)	237 (26.7)	
Post-secondary/tertiary	359 (46.2)	391 (44.0)	
Marital status			0.106
Married/partner	587 (77.1)	651 (73.6)	
Divorced/separated/single	130 (17.1)	161 (18.2)	
Widowed	44 (5.8)	73 (8.3)	
Annual personal income (New Zealand dollar)			<0.001
0-25,000	207 (31.1)	282 (38.0)	
25,001-50,000	241 (36.2)	282 (38.0)	
50,001-70,000	103 (15.5)	102 (13.8)	
>70,000	114 (17.1)	76 (10.2)	
Ethnicity			0.290
Non-Māori (European/others)	477 (62.6)	529 (60.1)	
Māori	285 (37.4)	352 (40.0)	
Current smoker			0.961
No	414 (93.7)	467 (93.6)	
Yes	28 (6.3)	32 (6.4)	
Alcohol consumption			0.001
No	340 (44.1)	466 (52.4)	
Yes	1,431 (55.9)	424 (47.6)	
Body mass index (kg/m^2^)			0.040
<25.0	244 (38.0)	252 (33.1)	
25.0-29.9	242 (37.6)	281 (36.9)	
≥30.0	157 (24.4)	229 (30.1)	
Physical activity			0.090
Insufficient	86 (11.5)	125 (14.3)	
Sufficient	663 (88.5)	748 (85.7)	
Hypertension			<0.001
No	528 (71.3)	529 (61.0)	
Yes	213 (28.7)	338 (39.0)	

Values are presented as number (%).

**Table 2.** Predictors of the incidence of multimorbidity among adults 55 years and over, New Zealand 2006-2016

Characteristics	aOR (95 % CI)^1^	p-value
Age	1.02 (1.01, 1.04)	0.002
Sex		
Female	1.00 (reference)	
Male	1.00 (0.87, 1.15)	0.981
Ethnicity		
Non-Māori (European/others)	1.00 (reference)	
Māori	1.21 (1.05, 1.40)	0.010
Education		
No secondary	1.00 (reference)	
Secondary	0.91 (0.76, 1.10)	0.353
Post-secondary/tertiary	0.84 (0.71, 0.99)	0.043
Income (New Zealand dollar)		
<20,000	1.00 (reference)	
25,001-50,000	0.85 (0.67, 1.09)	0.212
>50,000	0.72 (0.54, 0.95)	0.023
Marital status		
Married/ partner	1.00 (reference)	
Separated/divorced/single/widowed	1.13 (1.01, 1.28)	0.046
Current smoker (yes)	1.17 (0.91, 1.50)	0.230
Regular alcohol consumption (yes)	0.80 (0.69, 0.94)	<0.001
Adequate physical activity (yes)	0.84 (0.76, 0.92)	<0.001
Body mass index (kg/m^2^)		
<25.0	1.00 (reference)	
25.0-29.9	1.09 (0.91, 1.32)	0.348
≥30.0	1.48 (1.23, 1.79)	<0.001
Hypertension (yes)	1.46 (1.26, 1.69)	<0.001
One chronic condition at baseline	3.13 (2.62, 3.73)	<0.001

aOR, adjusted odds ratio; CI, confidence interval.

1 Adjusted for age and sex.

## References

[1] Marengoni A, Angleman S, Melis R, Mangialasche F, Karp A, Garmen A (2011). Aging with multimorbidity: a systematic review of the literature. Ageing Res Rev.

[2] Marengoni A, von Strauss E, Rizzuto D, Winblad B, Fratiglioni L (2009). The impact of chronic multimorbidity and disability on functional decline and survival in elderly persons. A community-based, longitudinal study. J Intern Med.

[3] McPhail SM (2016). Multimorbidity in chronic disease: impact on health care resources and costs. Risk Manag Healthc Policy.

[4] Fortin M, Lapointe L, Hudon C, Vanasse A, Ntetu AL, Maltais D (2004). Multimorbidity and quality of life in primary care: a systematic review. Health Qual Life Outcomes.

[5] France EF, Wyke S, Gunn JM, Mair FS, McLean G, Mercer SW (2012). Multimorbidity in primary care: a systematic review of prospective cohort studies. Br J Gen Pract.

[6] Ryan A, Wallace E, O’Hara P, Smith SM (2015). Multimorbidity and functional decline in community-dwelling adults: a systematic review. Health Qual Life Outcomes.

[7] Nunes BP, Flores TR, Mielke GI, Thumé E, Facchini LA (2016). Multi-morbidity and mortality in older adults: a systematic review and meta-analysis. Arch Gerontol Geriatr.

[8] Fortin M, Stewart M, Poitras ME, Almirall J, Maddocks H (2012). A systematic review of prevalence studies on multimorbidity: toward a more uniform methodology. Ann Fam Med.

[9] Melis R, Marengoni A, Angleman S, Fratiglioni L (2014). Incidence and predictors of multimorbidity in the elderly: a population-based longitudinal study. PLoS One.

[10] Ruel G, Shi Z, Zhen S, Zuo H, Kröger E, Sirois C (2014). Association between nutrition and the evolution of multimorbidity: the importance of fruits and vegetables and whole grain products. Clin Nutr.

[11] Wikström K, Lindström J, Harald K, Peltonen M, Laatikainen T (2015). Clinical and lifestyle-related risk factors for incident multimorbidity: 10-year follow-up of Finnish population-based cohorts 1982-2012. Eur J Intern Med.

[12] Dhalwani NN, O’Donovan G, Zaccardi F, Hamer M, Yates T, Davies M (2016). Long terms trends of multimorbidity and association with physical activity in older English population. Int J Behav Nutr Phys Act.

[13] Dhalwani NN, Zaccardi F, O’Donovan G, Carter P, Hamer M, Yates T (2017). Association between lifestyle factors and the incidence of multimorbidity in an older English population. J Gerontol A Biol Sci Med Sci.

[14] Sullivan KM, Dean A, Soe MM (2009). On academics: OpenEpi: a web-based epidemiologic and statistical calculator for public health. Public Health Rep.

[15] Roberts KC, Rao DP, Bennett TL, Loukine L, Jayaraman GC (2015). Prevalence and patterns of chronic disease multimorbidity and associated determinants in Canada. Health Promot Chronic Dis Prev Can.

[16] Nagel G, Peter R, Braig S, Hermann S, Rohrmann S, Linseisen J (2008). The impact of education on risk factors and the occurrence of multimorbidity in the EPIC-Heidelberg cohort. BMC Public Health.

[17] Tucker-Seeley RD, Li Y, Sorensen G, Subramanian SV (2011). Lifecourse socioeconomic circumstances and multimorbidity among older adults. BMC Public Health.

[18] Forechi L, Mill JG, Griep RH, Santos I, Pitanga F, Molina MD (2018). Adherence to physical activity in adults with chronic diseases: ELSA-Brasil. Rev Saude Publica.

[19] Pham TT, Callinan S, Livingston M (2019). Patterns of alcohol consumption among people with major chronic diseases. Aust J Prim Health.

[20] Shield KD, Parry C, Rehm J (2013). Chronic diseases and conditions related to alcohol use. Alcohol Res.

[21] Sarkar C, Dodhia H, Crompton J, Schofield P, White P, Millett C (2015). Hypertension: a cross-sectional study of the role of multi-morbidity in blood pressure control. BMC Fam Pract.

