# Supplementary material for: Socio-demographic and lifestyle factors associated with multimorbidity in New Zealand
Source: Epidemiol Health. 2019 Dec 27;42:e2020001. doi: 10.4178/epih.e2020001 (PMC7005455; doi:10.4178/epih.e2020001)
Supplement: Supplementary file 1 [file epih-42-e2020001-suppl.docx]

Wave (1), 2008

N=2474

Wave (2), 2010

N=1981

Wave (3), 2012

N=1860

Wave (5), 2016

N=1563

***Loss to follow up***

Excluded MM (N=957)

Incidence

Modelling

Participants without or with one disease (N=1673)

Number of new cases of MM /wave

Predictors of MM incidence over time

At risk population

No disease (N=776)

One disease (N=897)

Total number of new cases of MM (N=590)

Wave (3), 2014

N=1688

 Death =6

Other reason=152

Death =82

Other reason=411

Death =62

Other reason=59

Death =65

Other reason=107

Death =17

Other reason=108

**Supplementary Material 1. Study flowchart.**
